# Supplementary material for: Antiviral Mechanisms of N-Phenyl Benzamides on Coxsackie Virus A9
Source: Pharmaceutics. 2023 Mar 22;15(3):1028. doi: 10.3390/pharmaceutics15031028 (PMC10058015; doi:10.3390/pharmaceutics15031028)
Supplement: Supplementary file 1 [file pharmaceutics-15-01028-s001.zip › pharmaceutics-2253913-supplementary.pdf]

Supplementary material: Antiviral mechanisms of N-phenyl benzamides on Coxsackie virus A9

Mira Laajala[1], Kerttu Kalander[1], Sara Consalvi[2], Olivier Sheik Amamuddy[3], Özlem Tastan Bishop[3], Mariangela Biava[2], Giovanna Poce[2], Varpu Marjomäki[1*]

1. Department of Biological and Environmental Science/Nanoscience Center, University of Jyväskylä, 40014 Jyväskylä, Finland
2. Department of Chemistry and Technologies of Drug, Sapienza University of Rome, piazzale A. Moro 5, 00185 Rome, Italy
3. Research Unit in Bioinformatics (RUBi), Department of Biochemistry and Microbiology, Rhodes University, Makhanda 6140, South Africa

* Correspondence: varpu.s.marjomaki@jyu.fi; Tel.: +358405634422

In-house chemical library

Our in-house compound library is composed of 200 compounds of which 126 are pyrroles of general formula I, 23 pyrazoles of general formula II, 5 imidazoles of general formula III, 13 *N*-phenyl benzamides of general formula IV, 4 benzamines of general formula V, 4 phenyl cinnamamides of general formula VI, 2 methanimines of general formula VII, 4 triazoles of general formula VIII, 9 F-anilines of general formula IX, 9 phenylhydrazides of general formula X, and 1 furane XI as reported in figure S1.

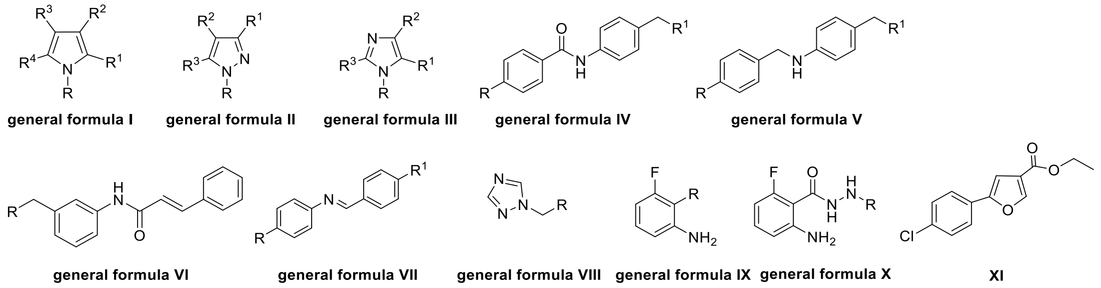

Figure S1. General formulas of compounds used for the screening.

The more detailed chemical structures of the molecules (CL1-CL220) are presented in table S1.

Table S1. Chemical structures of CL1-CL220 molecules.

**Compound**	Chemical structure
**CL1**	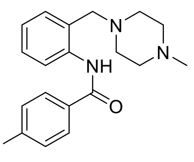

**CL2**	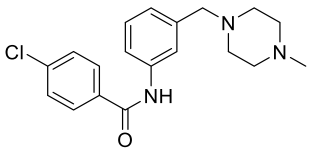
**CL3**	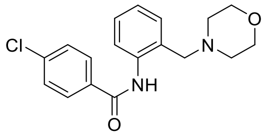
**CL4**	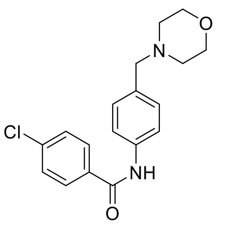
**CL5**	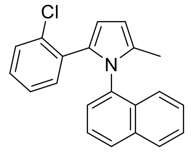
**CL6**	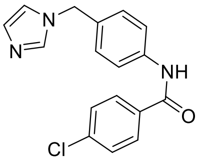
**CL7**	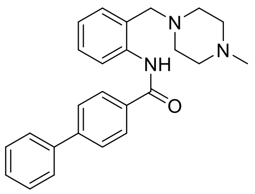
**CL8**	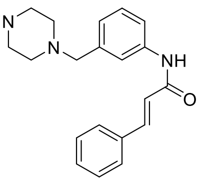
**CL9**	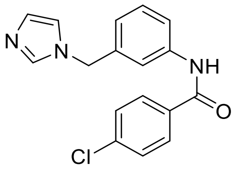
**CL10**	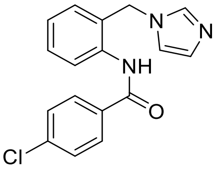

**CL11**	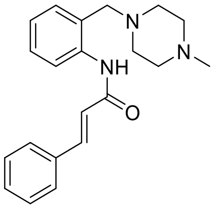
**CL12**	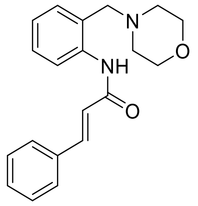
**CL13**	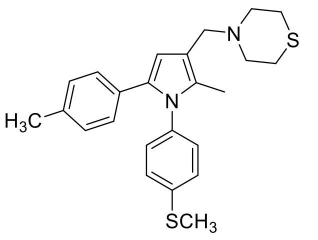
**CL14**	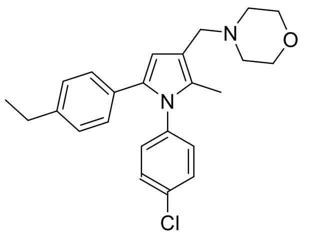
**CL15**	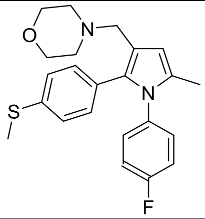
**CL16**	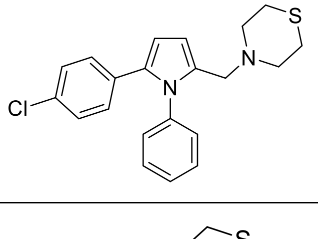
**CL17**	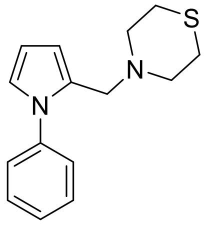

**CL20**	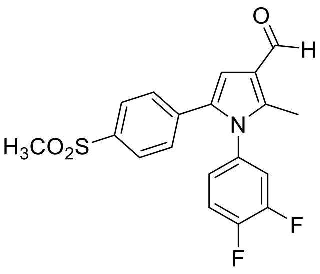
**CL21**	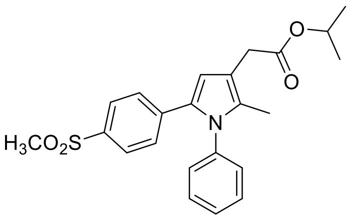
**CL22**	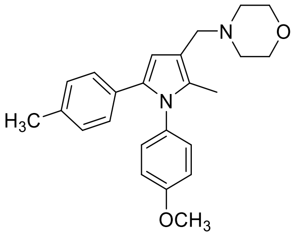
**CL23**	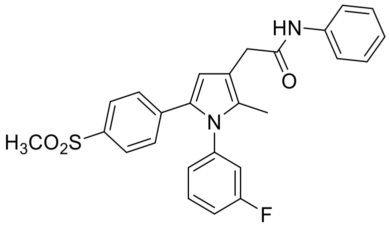
**CL24**	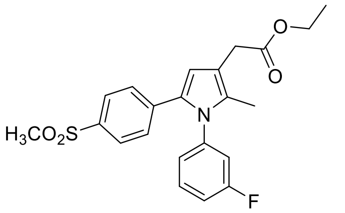
**CL25**	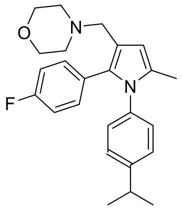
**CL26**	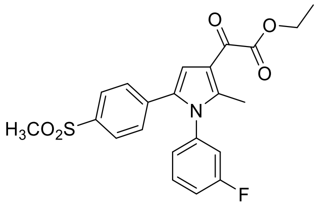

**CL27**	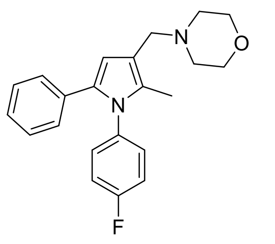
**CL28**	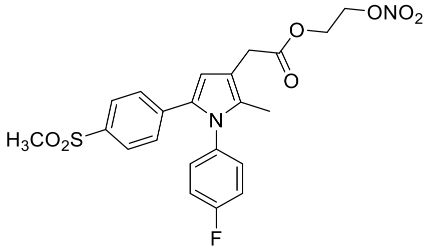
**CL29**	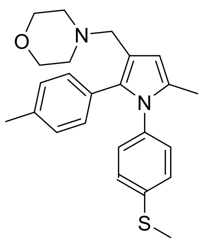
**CL30**	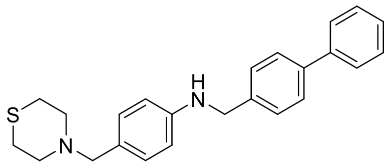
**CL31**	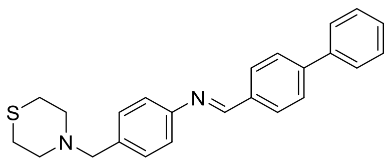
**CL32**	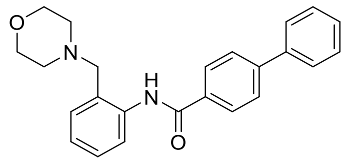
**CL33**	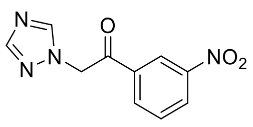
**CL34**	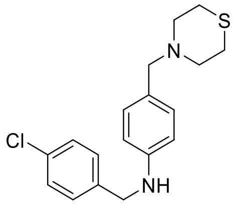
**CL35**	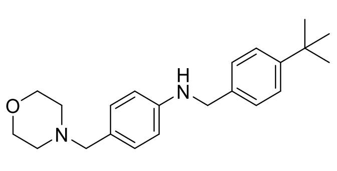

**CL37**	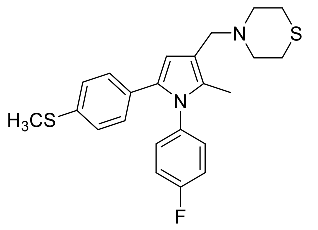
**CL38**	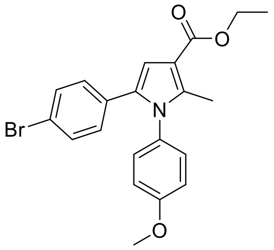
**CL39**	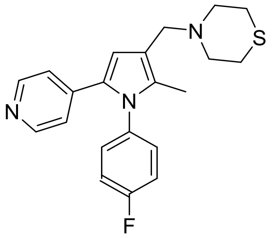
**CL40**	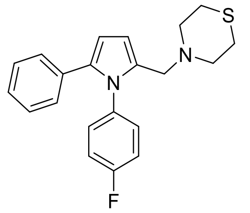
**CL41**	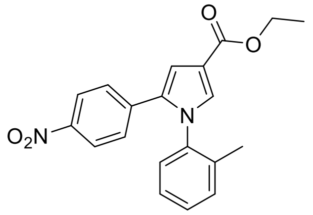
**CL42**	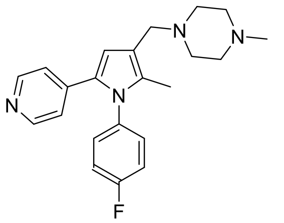
**CL43**	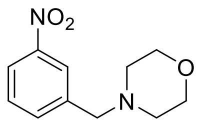
**CL44**	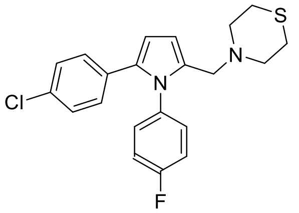

**CL45**	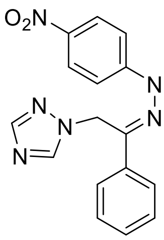
**CL46**	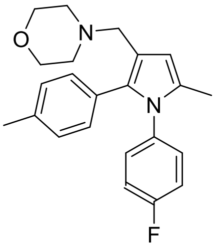
**CL47**	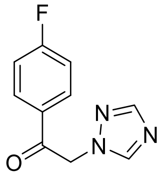
**CL48**	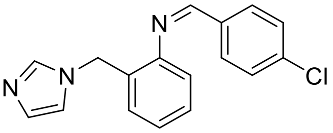
**CL49**	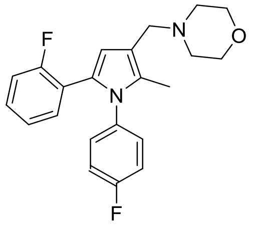
**CL50**	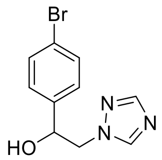
**CL51**	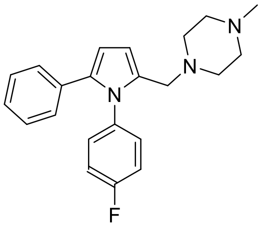
**CL52**	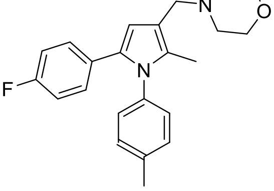

**CL53**	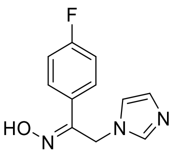
**CL54**	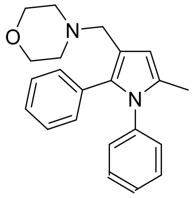
**CL55**	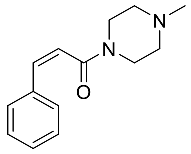
**CL56**	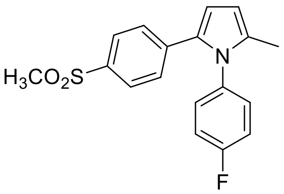
**CL57**	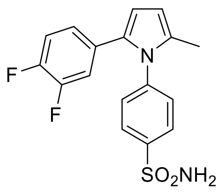
**CL58**	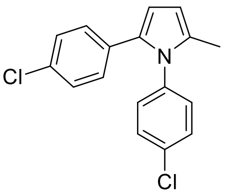
**CL59**	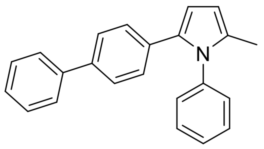
**CL60**	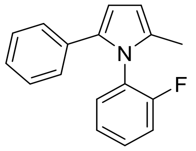
**CL61**	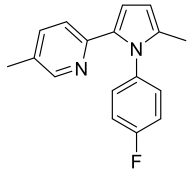

**CL62**	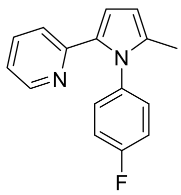
**CL63**	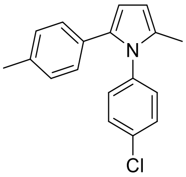
**CL64**	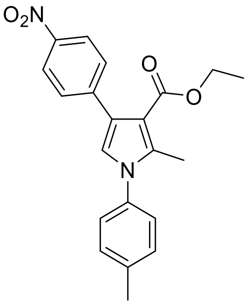
**CL65**	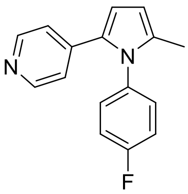
**CL66**	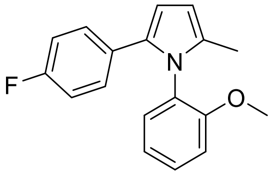
**CL67**	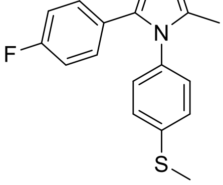
**CL68**	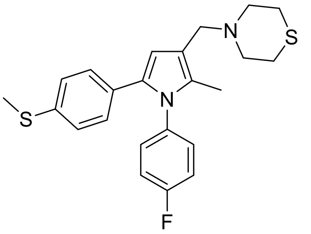

**CL69**	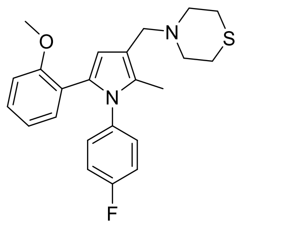
**CL70**	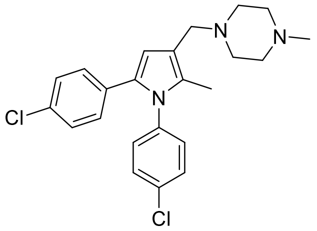
**CL71**	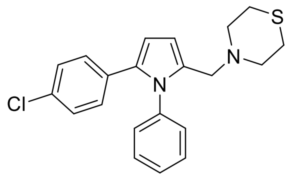
**CL72**	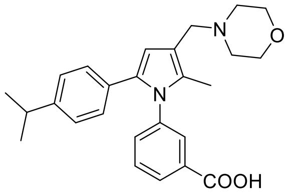
**CL73**	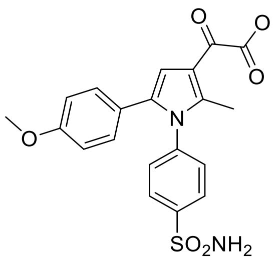
**CL74**	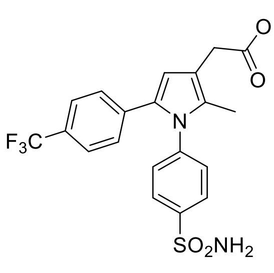
**CL76**	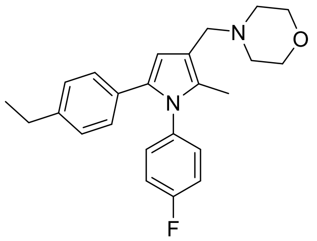

**CL78**	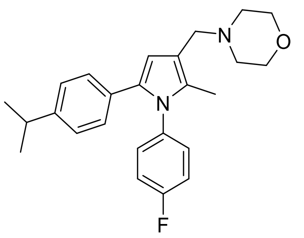
**CL79**	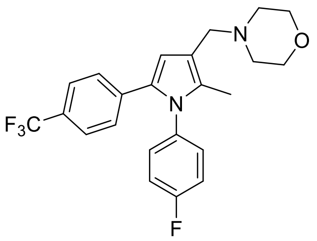
**CL80**	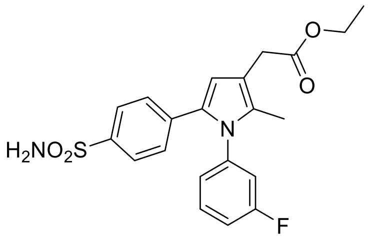
**CL81**	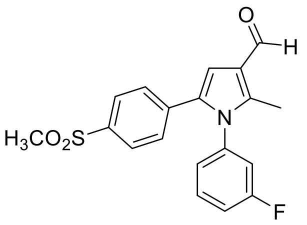
**CL82**	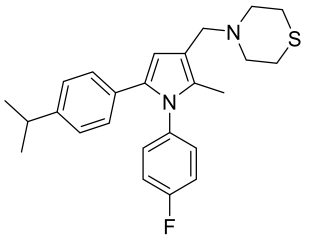
**CL83**	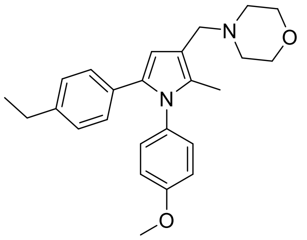
**CL84**	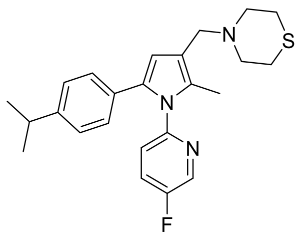

**CL85**	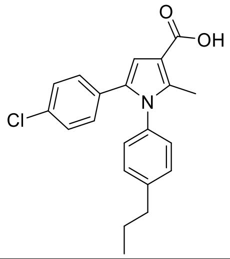
**CL86**	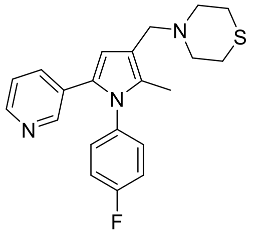
**CL87**	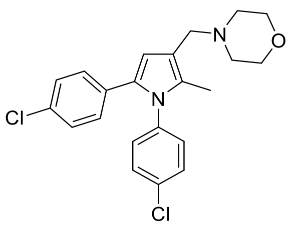
**CL88**	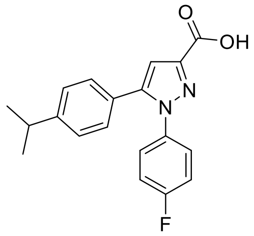
**CL89**	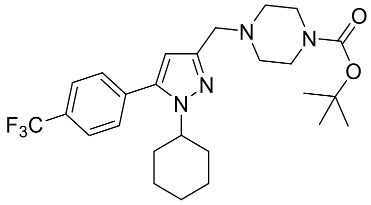
**CL90**	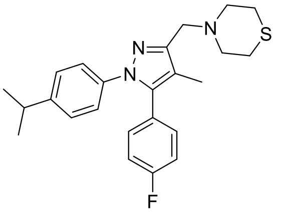
**CL91**	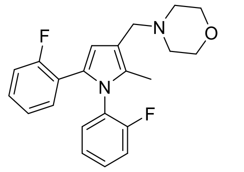

**CL92**	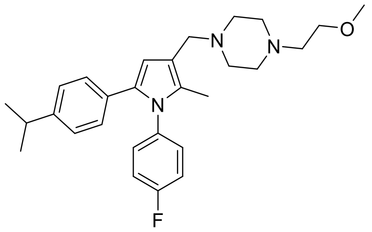
**CL93**	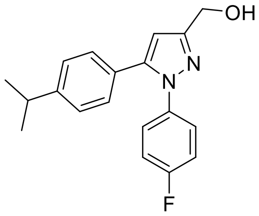
**CL94**	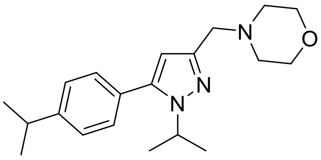
**CL95**	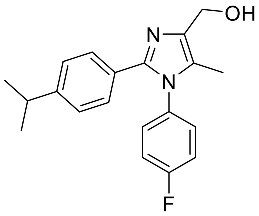
**CL96**	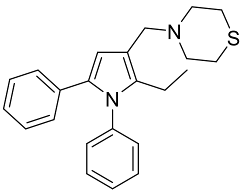
**CL97**	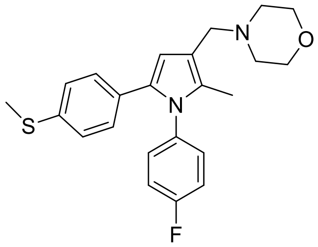
**CL98**	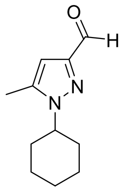

**CL99**	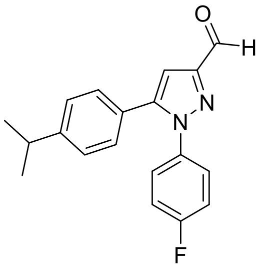
**CL100**	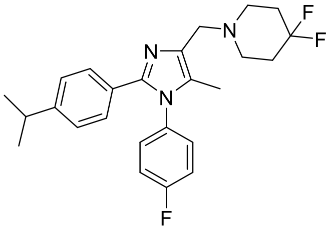
**CL101**	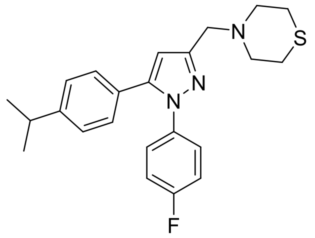
**CL102**	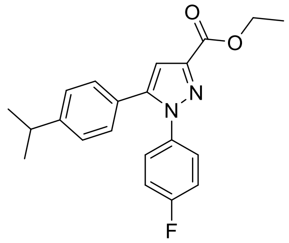
**CL103**	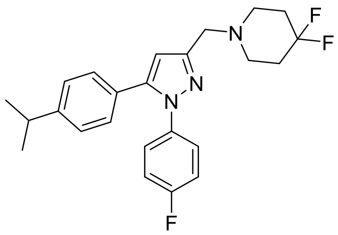
**CL104**	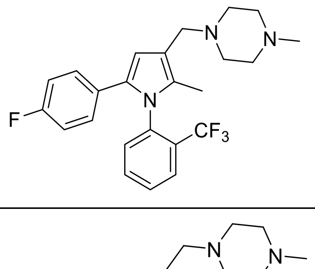
**CL105**	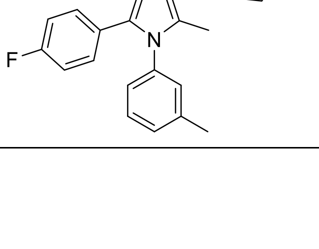

**CL106**	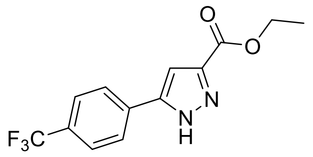
**CL107**	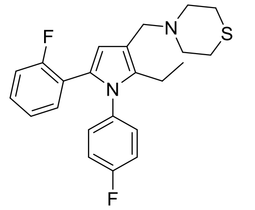
**CL109**	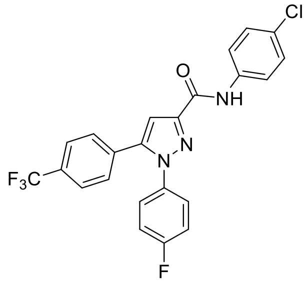
**CL110**	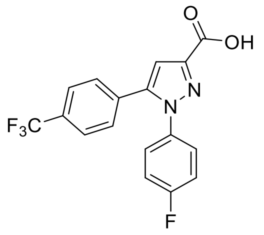
**CL111**	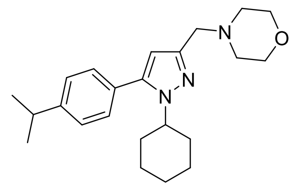
**CL112**	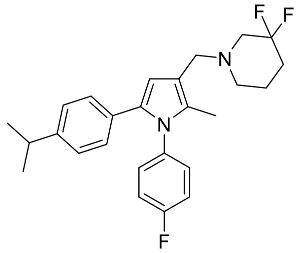
**CL113**	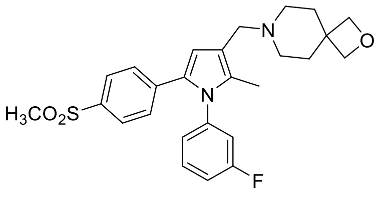

**CL114**	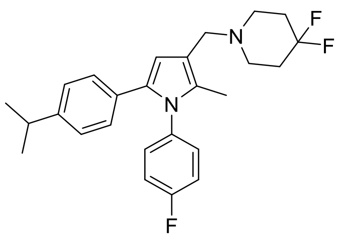
**CL115**	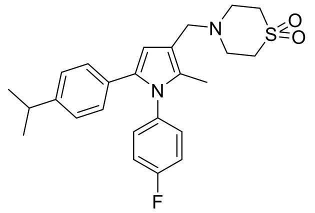
**CL116**	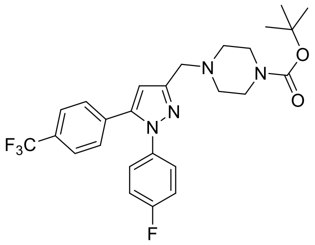
**CL117**	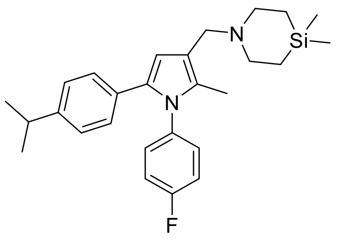
**CL118**	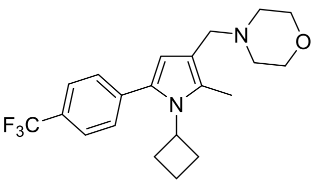
**CL119**	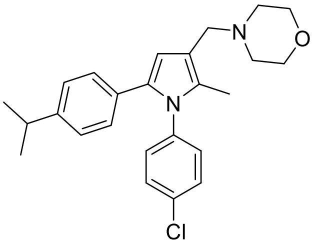
**CL120**	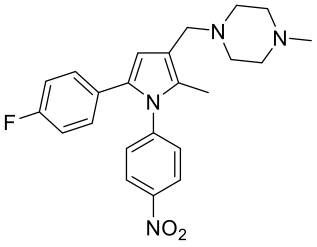

**CL123**	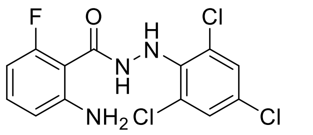
**CL124**	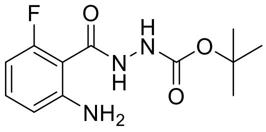
**CL125**	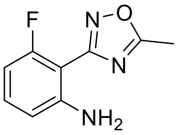
**CL126**	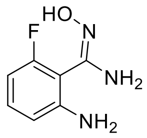
**CL127**	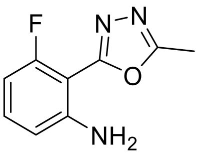
**CL129**	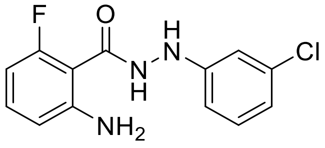
**CL131**	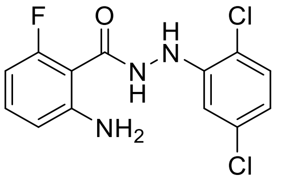
**CL133**	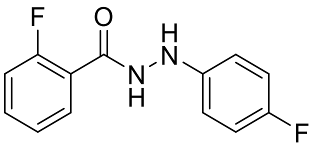
**CL134**	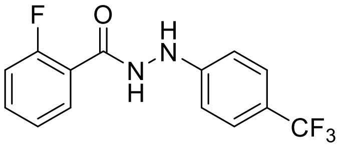
**CL135**	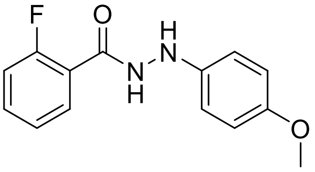

**CL136**	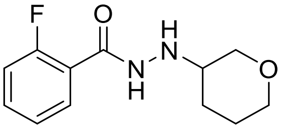
**CL137**	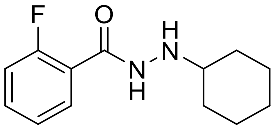
**CL138**	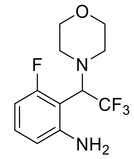
**CL140**	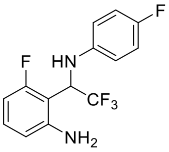
**CL141**	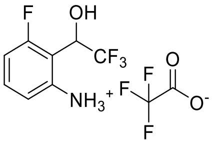
**CL142**	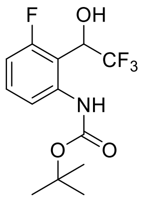
**CL145**	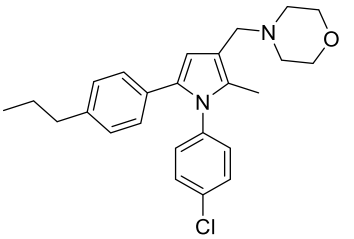
**CL146**	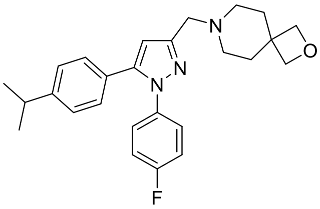
**CL147**	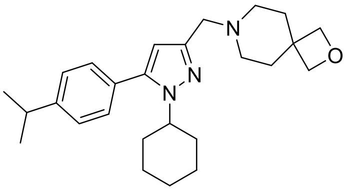

**CL148**	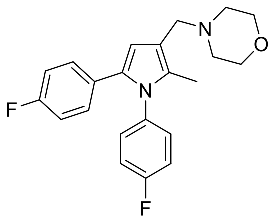
**CL149**	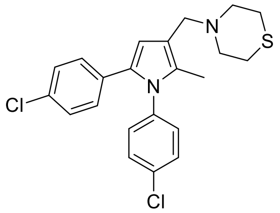
**CL150**	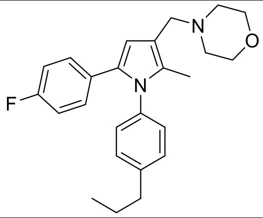
**CL151**	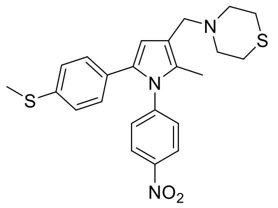
**CL152**	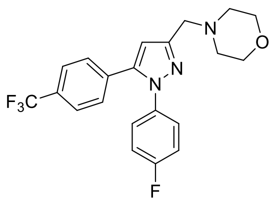
**CL153**	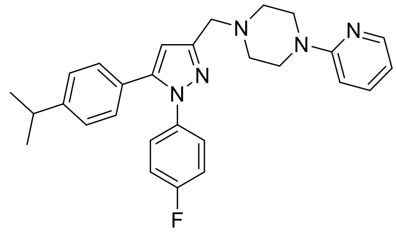
**CL154**	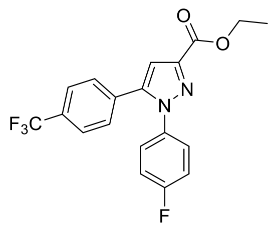

**CL155**	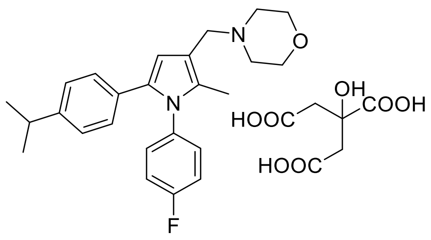
**CL157**	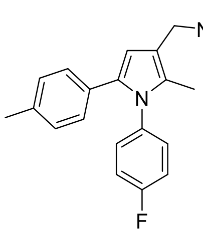
**CL158**	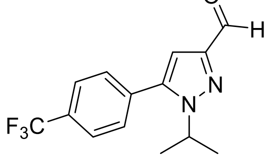
**CL159**	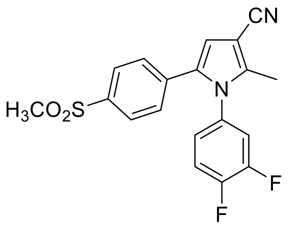
**CL161**	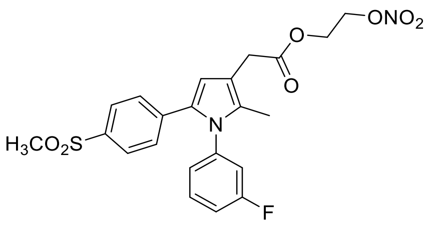
**CL162**	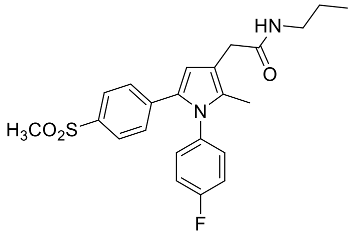
**CL163**	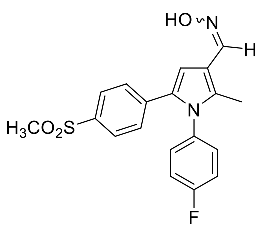

**CL164**	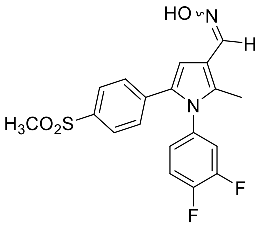
**CL166**	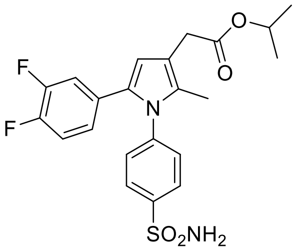
**CL167**	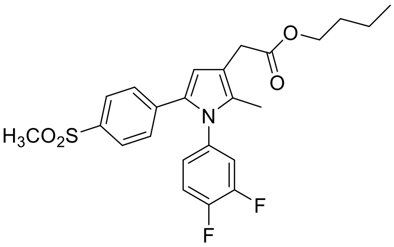
**CL169**	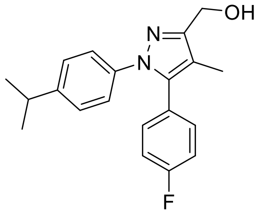
**CL170**	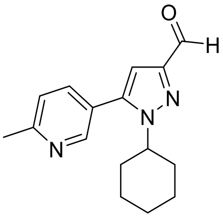
**CL171**	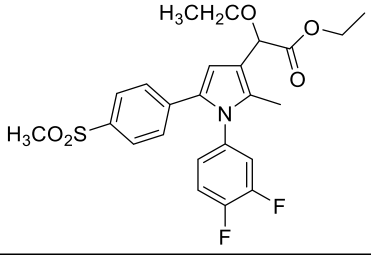
**CL172**	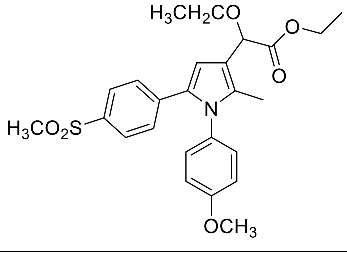

**CL173**	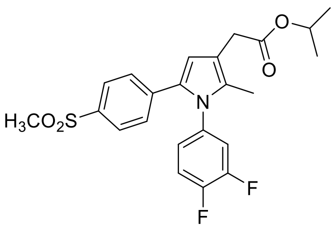
**CL174**	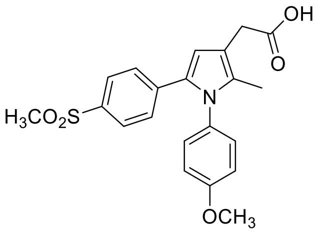
**CL175**	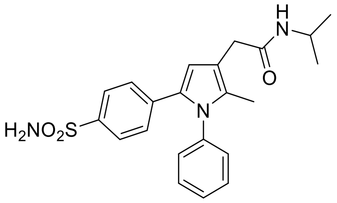
**CL176**	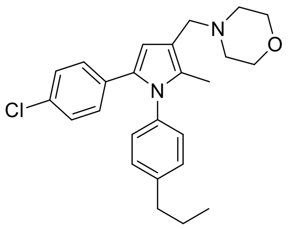
**CL177**	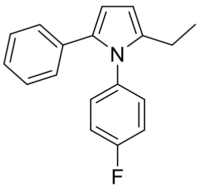
**CL178**	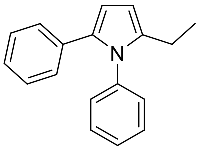
**CL179**	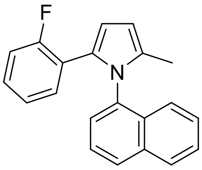
**CL180**	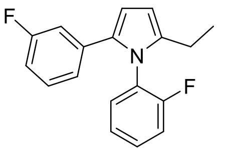

**CL181**	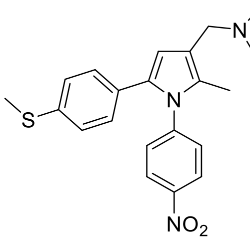
**CL182**	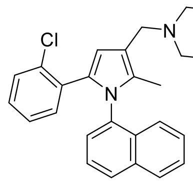
**CL183**	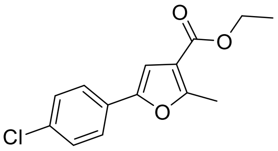
**CL184**	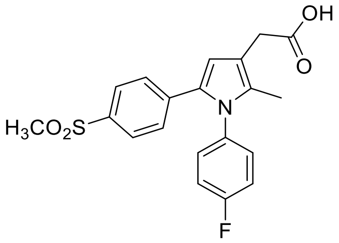
**CL185**	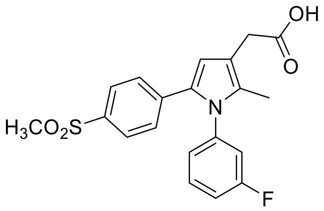
**CL186**	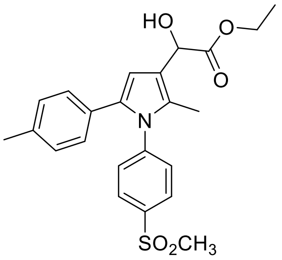
**CL187**	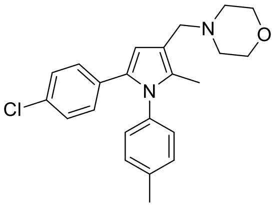
**CL188**	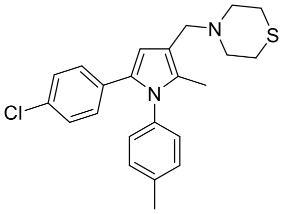

**CL189**	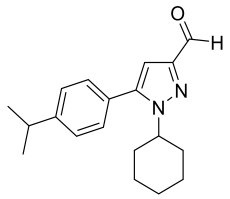
**CL190**	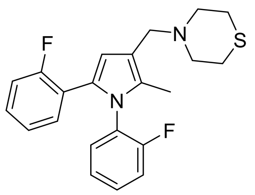
**CL191**	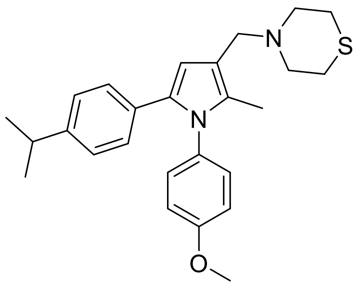
**CL192**	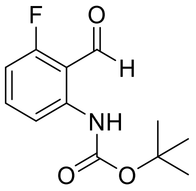
**CL193**	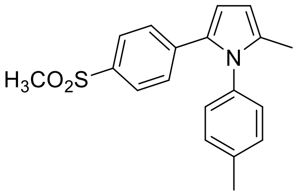
**CL194**	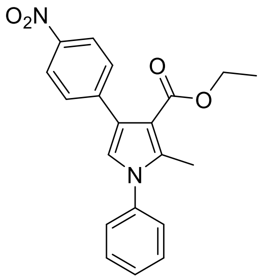
**CL195**	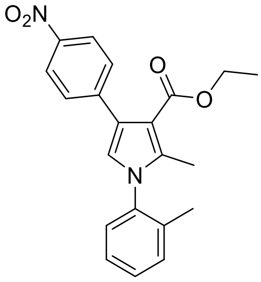

**CL196**	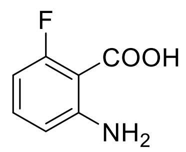
**CL197**	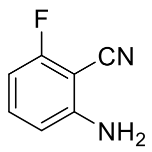
**CL199**	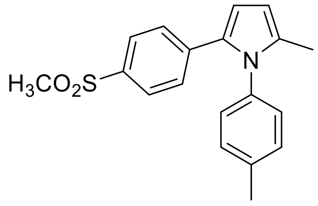
**CL200**	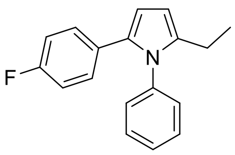
**CL201**	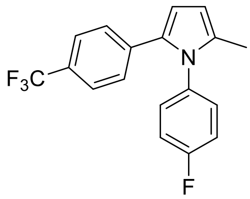
**CL202**	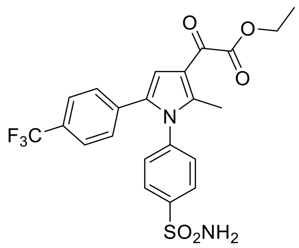
**CL203**	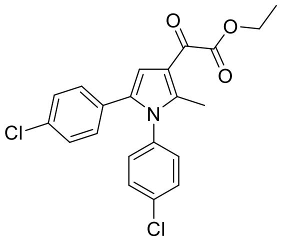
**CL204**	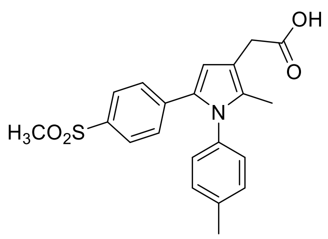

**CL205**	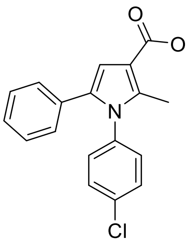
**CL208**	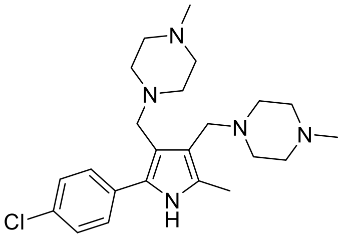
**CL209**	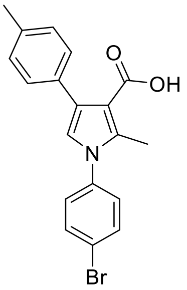
**CL210**	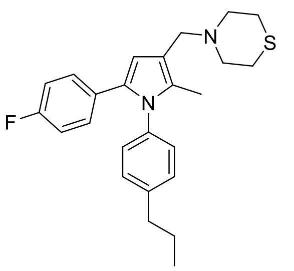
**CL211**	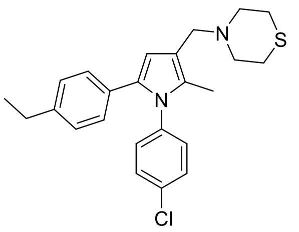
**CL214**	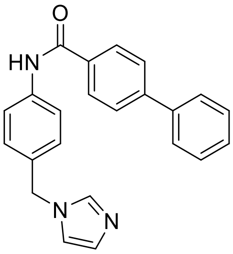

**CL215**	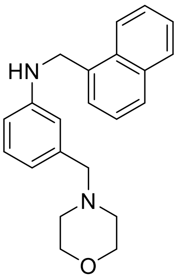
**CL216**	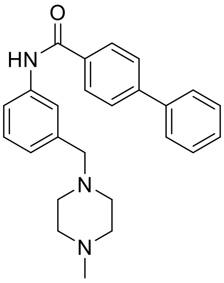
**CL217**	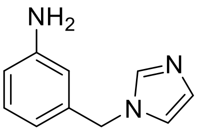
**CL218**	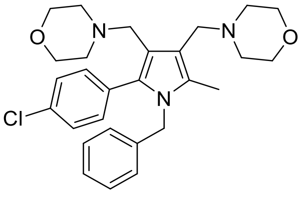
**CL219**	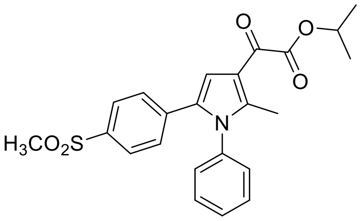
**CL220**	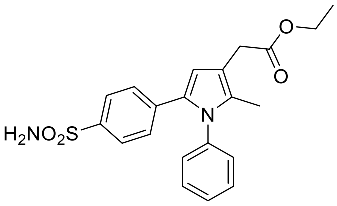

Chemistry

Reagents and solvents were obtained from commercial sources (Sigma-Aldrich, Activate Scientific). Analytical TLC was performed on Merck silica gel (60F254) precoated plates (0.25 mm). The compounds were visualized under UV light (254 nm) and/or stained with the relevant reagent. Flash column chromatography was performed on silica gel with pore size 60 Å, 230–400 mesh particle size, and 40–63 μm particle size, with the indicated solvents. The yields refer to the purified products, and they were not optimized. All the solid compounds were obtained as amorphous solids, and melting points were not measured. ^{1}H NMR spectra were recorded on a Bruker Avance III NMR spec-

trometer and in a Bruker DPX Avance 400 MHz instrument equipped with a QNP probe and are reported in ppm using tetramethylsilane as internal standard. ^{13}C NMR spectra were recorded on a Bruker Avance III NMR spectrometer at 295 K and are reported in ppm using solvent as internal standard ($CDCl_3$ at 77.0 ppm). Mass spectra data measurements were performed on a VG-Analytical Autospec Q mass spectrometer. Analytical purity was ≥95% unless stated otherwise. The purities of the final compounds were checked using a Waters ZQ2000 coupled with LC Waters 2795 and Waters 2996 PDA detector.

CL212 and CL213 were prepared according to the reaction pathway reported in Scheme S1. Briefly, the coupling reaction between the 4-((4-methylpiperazin-1-yl)methyl)aniline and the appropriate benzoic acids **1** and **2** in the presence of HATU, DIPEA in dry DMF afforded CL212 and CL213 in good yields.

Scheme S1. Synthetic pathway for CL212 and CL213.

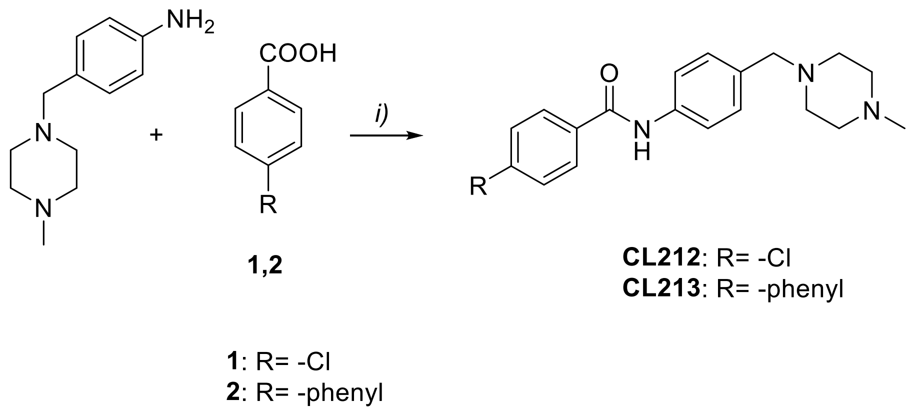

Reagents and conditions: i) HATU, DIPEA, DMF, rt, overnight, 50-95%.

General Procedures

To a solution of 4-((4-methylpiperazin-1-yl)methyl)aniline (0.974 mmol) in anhydrous DMF (3 ml), HATU (1.12 mmol) and DIPEA (4.87 mmol, 0.84 ml) were added under N_2. The obtained mixture was stirred for 30 min at room temperature, and then the appropriate benzoic acid (**1**,**2**) (1.07 mmol) was added. The resulting mixture was stirred for 18 h at room temperature and at the end quenched with 10 ml of saturated $NaHCO_3$ solution. The mixture was extracted with ethylacetate, washed with brine, dried over Na_2SO_4, and concentrated in vacuo. The crude material was purified by column chromatography (DCM/MeOH) 20/1 (v/v)) and then crystallized from DCM.

4-chloro-*N*-(4-((4-methylpiperazin-1-yl)methyl)phenyl)benzamide (CL212). White solid (yield 50%). ^1H NMR (400 MHz, $CDCl_3$): δ ppm= 7.83 (d, 2H), 7.75 (s broad, 1H), 7.58 (d, 2H), 7.49 (d, 2H), 7.35 (d, 2H), 3.51 (s, 2H), 2.48 (s broad, 8H), 2.30 (s, 3H). ^{13}C NMR (100 MHz, $CDCl_3$): δ ppm= 163.82, 136.44, 135.82, 134.01, 133.75, 129.85, 129.68, 128.21, 122.19, 66.75, 55.55, 53.56, 43.90. LRMS $(M + H)^+$ (ESI+) 344.22 $[M + H]^+$(calcd for $C_{19}H_{22}ClN_3O$ 343.15).

***N*-(4-((4-methylpiperazin-1-yl)methyl)phenyl)-[1,1'-biphenyl]-4-carboxamide (CL213).** White solid (yield 95%). ^1H NMR (400 MHz, $CDCl_3$): δ ppm= 7.97 (d, 2H), 7.85 (s

broad, 1H), 7.74 (d, 2H), 7.65 (m, 4H), 7.50 (t, 2H), 7.42 (tt, 1H), 7.36 (d, 2H), 3.52 (s, 2H), 2.50 (s broad, 8H), 2.31 (s, 3H). ^{13}C NMR (100 MHz, $CDCl_3$): δ ppm= 168.82, 144.50, 138.63, 137.54, 135.75, 134.85, 130.78, 129.21, 128.95, 127.98, 127.59, 127.19, 119.91, 61.75, 53.85, 52.56, 45.20. LRMS $(M + H)^+$(ESI+) 386.42 $[M + H]^+$(calcd for $C_{25}H_{27}N_3O$ 385.22).

Table S2. Physicochemical properties, lipophilicity, solubility, pharmacokinetics, drug likeness and medicinal chemistry of CL212 and CL213 predicted using SwissADME.

**Compound**	**CL213**	**CL212**
**Physicochemical properties**		
Formula	$C_{25}H_{27}N_3O$	$C_{19}H_{22}ClN_3O$
Molecular weight	385.50 g/mol	343.85 g/mol
Num. heavy atoms	29	24
Num. arom. heavy atoms	18	12
Fraction Csp3	0.24	0.32
Num. rotatable bonds	6	5
Num. H-bond acceptors	3	3
Num. H-bond donors	1	1
Molar Refractivity	126.60	106.18
TPSA	35.58 Å^2	35.58 Å^2
**Lipophilicity**		
Log $P_{o/w}$ (iLOGP)	3.86	3.45
Log $P_{o/w}$ (XLOGP3)	4.03	3.03
Log $P_{o/w}$ (WLOGP)	3.25	2.24
Log $P_{o/w}$ (MLOGP)	3.45	2.87
Log $P_{o/w}$ (SILICOS-IT)	4.12	3.17
Consensus Log $P_{o/w}$	3.74	2.95
og S (ESOL)	-4.83	3.92
Solubility	5.67e-03 mg/ml ; 1.47e-05 mol/l	4.13e-02 mg/ml ; 1.20e-04 mol/l
Class	Moderately soluble	Soluble
Log S (Ali)	-4.48	-3.44
Solubility	1.28e-02 mg/ml ; 3.31e-05 mol/l	1.24e-01 mg/ml ; 3.61e-04 mol/l
Class	Moderately soluble	Soluble
Log S (SILICOS-IT)	-7.94	-6.07
Solubility	4.44e-06 mg/ml ; 1.15e-08 mol/l	2.93e-04 mg/ml ; 8.53e-07 mol/l
Class	Poorly soluble	Poorly soluble
**Pharmacokynetics**		
GI absorption	High	High
BBB permeant	Yes	Yes
P-gp substrate	Yes	No
CYP1A2 inhibitor	Yes	Yes
CYP2C19 inhibitor	No	Yes

CYP2C9 inhibitor	No	No
CYP2D6 inhibitor	Yes	Yes
CYP3A4 inhibitor	Yes	Yes
Log K_p (skin permeation)	-5.79 cm/s	-6.25 cm/s
**Druglikeness**		
Lipinski	Yes; 0 violation	Yes; 0 violation
Ghose	Yes	Yes
Veber	Yes	Yes
Egan	Yes	Yes
Muegge	Yes	Yes
Bioavailability Score	0.55	0.55
**Medicinal Chemistry**		
PAINS	0 alert	0 alert
Brenk	0 alert	0 alert
Leadlikeness	No; 2 violations: MW>350, XLOGP3>3.5	Yes
Synthetic accessibility	2.55	2.11

Antiviral activity

The antiviral activity of the 200 molecules (CL1-CL220) was studied using a cytopathic effect (CPE) assay. The molecules were divided into four different 96-well plates, and in addition, the plates contained cell controls, virus controls and inhibitor controls. The inhibitor controls were 10 μM epigallocatechin gallate (EGCG, Sigma) and 10 μM pleconaril (Sigma). In the first screen (Figure S2, left panel) CVA9 (1.6×10^7 PFU/ml) was incubated for 1 h at +37 °C with 10 μM molecules (CL1-CL220) diluted in 2 mM $MgCl_2$/PBS. Next, the virus/molecule mixture was diluted 10X in DMEM supplemented with 1% FBS and glutamax to obtain MOI 8 and 1 μM molecule concentration on cells. The infection was allowed to proceed in cells at +37 °C for 48 h. The second screen (Figure S2, right panel) was carried out with small adjustments in the protocol and included only molecules CL169-CL220 as they showed the most potential in the first screen. CVA9 (1.6×10^7 PFU/ml) was incubated for 1 h at +37 °C with 10 μM molecules diluted directly in DMEM supplemented with 1% FBS and glutamax. The virus/molecule mixture was then directly added on cells without further dilution to obtain MOI 80 and 10 μM molecule concentration on cells. The infection was allowed to proceed in cells at +37 °C for 24 h. After 24 or 48 h, the cells were washed with PBS and then stained with a crystal violet containing dye (0.03% crystal violet, 2% ethanol and 3% formalin in water). Next, the excess stain was washed with sterile water and finally lysis buffer (47% ethanol, 12.5 mM HCl and 19 mM sodium citrate) was added to homogenize the solution. Absorbance was measured at 570 nm with a multiplate reader (VictorTM X4, PerkinElmer, Waltham, USA). The data was normalized against the absorbance of the cell control which was set to 100%.

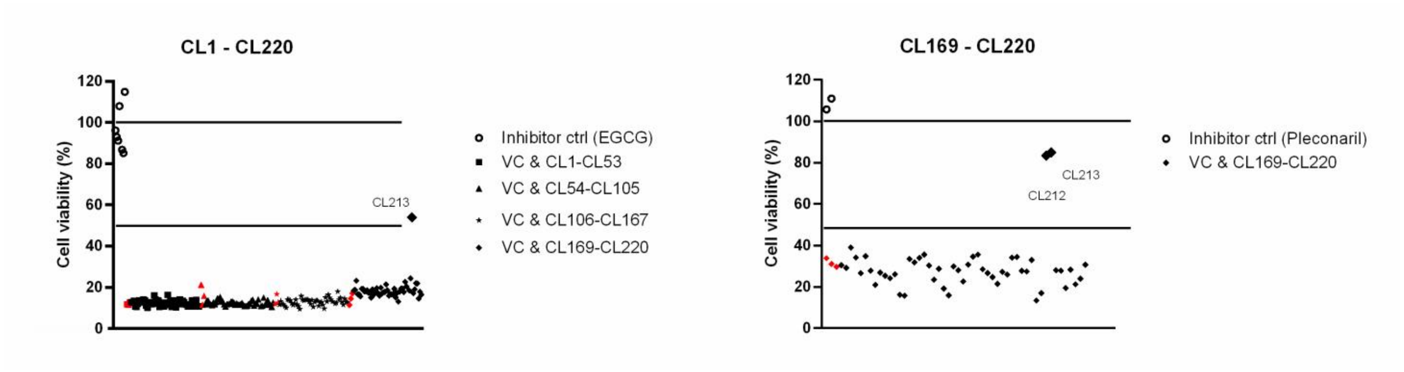

Figure S2. Antiviral activity of CL1-CL220 molecules against CVA9. The virus was incubated with the molecules for 1 h at +37 °C before adding the virus/molecule mixture on cells. In the left panel, MOI 8 and 48 h infection time was used, while in the right panel, MOI was 80 and infection time 24 h. The molecules were divided into four 96-well plates which are presented with different symbols. Each individual symbol represents one molecule or control. Virus controls (VC) are colored red and inhibitor controls epigallocatechin gallate (EGCG) or pleconaril are presented as open circles. Virus controls are presented before the molecules on the corresponding plate. Cell viability is normalized against the cell control which is set to 100%. Lines mark the 50% and 100% cell viabilities.
